# Incorporating bay leaf extract (*Laurus nobilis* L.) and determining the quality attributes of Turkish fermented sausage (sucuk)

**DOI:** 10.1002/fsn3.3929

**Published:** 2024-01-02

**Authors:** Hakan Benli, Pelin Şahin, Erdal Ağçam

**Affiliations:** ^1^ Department of Food Engineering, Faculty of Engineering Cukurova University Adana Turkey

**Keywords:** biogenic amines, natural antioxidant, refrigerated storage, traditional sausage, ultrasound‐assisted extraction

## Abstract

This study aimed at investigating the quality attributes of Turkish fermented sausage (sucuk) incorporated with bay leaf extract obtained as a natural antioxidant and a source of phytochemicals. Five different bay leaf extracts were obtained with distilled water and 60%, 70%, 80%, and 90% ethanol. The total phenolic contents and antioxidant activity values indicated that ultrasound‐assisted 70% ethanol extract was the most suitable extract. Furthermore, five groups of sucuks were manufactured with the addition of bay leaf extract (1, 5, and 10 mL/kg), ascorbic acid (500 mg/kg), and control. The extracts were produced similar pH values when compared to control and ascorbic acid samples. The treatments had no significant effect on moisture contents of sucuks. Bay leaf extracts produced comparable color, texture profile analysis, and TBARS values to control and ascorbic acid samples. Biogenic amine contents (mg/kg dry weight) of sucuks including tryptamine (6.43–30.66), 2‐phenylethylamine (2.24–32.04), putrescine (2.19–7.98), cadaverine (3.28–12.21), histamine (7.01–11.38), tyramine (3.27–71.07), spermidine (4.44–8.01), and spermine (53.96–68.25) were mostly within the lower ranges typically associated with sucuk. However, the lowest cadaverine values observed at the end of storage in the bay leaf extract added samples indicated that bay leaf extract might be effective in decreasing cadaverine values during storage. The addition of bay leaf extract caused similar sensory attributes to the control and ascorbic acid samples. This study revealed that Turkish fermented sucuks could be effectively incorporated with bay leaf extracts without a negative effect on the quality attributes or consumer acceptability.

## Graphical abstract

This study aimed at investigating the quality attributes of Turkish fermented sausage (sucuk) incorporated with bay leaf extract obtained as a natural antioxidant and a source of phytochemicals. This study revealed that Turkish fermented sucuks could be effectively incorporated with bay leaf extracts without a negative effect on the quality attributes or consumer acceptability.

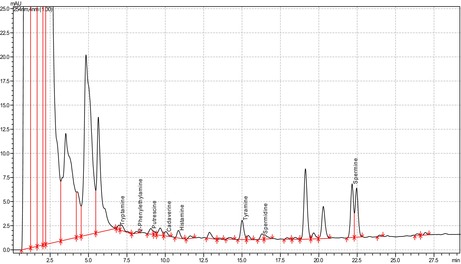

## 1 INTRODUCTION

Sucuk is a type of Turkish fermented sausage produced typically by mixing ground beef and fat and/or sheep tail fat with salt, nitrite or/and nitrate (cure), sugar, and some species comprising red pepper, black pepper, cumin, allspice, and garlic. Natural or synthetic casings are used for stuffing the sucuk dough. Sucuks are fermented by either inoculating a starter culture or by natural fermentation. The fermentation is followed by drying and ripening steps, which could be under natural or controlled climate conditions. The traditional method of producing Turkish sucuk mostly requires a drying period, and the end product is a semidry or dry sausage (Benli, 2017).

Biogenic amines are organic molecules with small molecular masses produced as a result of metabolic activity in microorganisms and can be found in various foods such as cheese, meat, wine, and vegetables. Biogenic amines with aromatic, aliphatic, and heterocyclic structures are produced through the decarboxylation of amino acids (Lorenzo et al., 2007; Spano et al., 2010; Wang et al., 2015). The formation of biogenic amines depends on many factors, the presence of free amino acids, the growth of microorganisms with high decarboxylase activity, and the existence of suitable conditions for decarboxylases (Lorenzo et al., 2020). Decarboxylase‐producing microorganisms remove the α‐carboxyl group of amino acids to generate corresponding biogenic amines (Wang, Liu, et al., 2022). Sun et al. (2020) reported that histamine, putrescine, cadaverine, tyramine, tryptamine, β‐phenylethylamine, spermidine, and spermine were among the most important biogenic amines in foods. Biogenic amines in foods are important for two reasons, the amount of biogenic amines, which is accepted as a quality indicator, and their potential toxic effects on human health (Ruiz‐Capillas & Herrero, 2019). Intake of higher levels of biogenic amines with foods might have toxic effects on cardiovascular and nervous systems and might cause arrhythmia and neurological headaches (Li et al., 2023). Amino acid decarboxylases are involved in a variety of biological processes, ranging from bacterial putrefaction to neurotransmission. Two types of decarboxylases in microbial cells were defined, including biodegradative decarboxylases and biosynthetic decarboxylases. The biodegradative decarboxylases were reported to cause biogenic amine formation during the putrefaction of meat. Biogenic amines were formed by numerous bacteria possessing amino acid decarboxylases under suitable conditions for enzymatic activity. Lee et al. (2018) reported that studies focused on two approaches for decreasing the formation of biogenic amines in the food matrix, including either inhibition of microbial growth or inhibition of the amino acid decarboxylase activity produced by microorganisms. However, limiting bacterial growth during fermentation might cause the fermented products to lose their authentic flavor. Alberto et al. (2007) indicated that putrescine formation was decreased by the presence of phenolic compounds, which are well‐known antioxidants, since the phenolic compounds might protect the cells from oxidative stress. Furthermore, the ethanol extracts of several spices were reported to have an inhibitory effect on the decarboxylase activity of microorganisms (Wendakoon & Sakaguchi, 1995). Natural antioxidant and antibacterial substances are important for the quality and safety of fermented sausages. In recent years, the use of spices and aromatic plants as preservatives has become widespread due to their natural antioxidant and antimicrobial properties (Zhang et al., 2017). Herbs and spices can be used whole or in extract form in meat and meat products (Yin & Cheng, 2003). The purpose of the extraction is to obtain the highest quality compounds with high antioxidant content. Phenolic‐based compounds found in plants can be isolated by various methods and used as natural antioxidants (Bozkurt, 2006; Dai & Mumper, 2010).

Lipid oxidation is one of the main factors that cause deterioration in the quality of meat and meat products. Fermented sausages could be susceptible to the lipid oxidation due to the high amount of fat in their composition and manufacturing processes like grinding (Manzoor et al., 2023). Synthetic antioxidants, such as butylated hydroxytoluene (BHT), butylated hydroxyanisole (BHA), and tertiary butylhydroquinone (TBHQ), could effectively reduce lipid oxidation. Since their usage was limited in meat products due to health concerns related to the toxicological effects of these synthetic additives, natural compounds, including plant extracts, could be used for decreasing lipid oxidation and food preservation (Bellucci et al., 2021; Manzoor et al., 2022).

Turkey is home to many plants with aromatic and medicinal properties due to its geographical location. Bay leaf (*Laurus nobilis* L.), as an evergreen large shrub, is one of the characteristic plants of the Mediterranean climate. Leaves can be collected from a mature plant all year round. Since fresh bay leaves have a pungent and bitter taste, they are usually left for drying to obtain a better and deeper flavor after harvesting. Bay leaf is consumed as a cooking ingredient and used in many traditional practices for various purposes (Batool et al., 2020). The bay leaf was reported to be rich with phytochemicals like phenolic compounds, including flavonoids such as quercetin, luteolin, apigenin, kaempferol, myrcetin derivatives, and flavan‐3‐ols (Dias et al., 2014; Muniz‐Marquez et al., 2013). Furthermore, Elmastas et al. (2006) reported that both water and ethanol extracts of bay leaf had strong total antioxidant activity and had effective reducing power, DPPH* free radical scavenging, superoxide anion radical scavenging, hydrogen peroxide scavenging, and metal chelating activities. In the literature, there are limited studies related to incorporating sausages with bay leaf extracts. Jia et al. (2020) studied the effects of incorporating seven spice extracts (0.3 g/kg) including bay leaf extract, into dry fermented mutton sausage. It was indicated that biogenic amine content was significantly decreased by spice extracts, while cassia and fennel extracts were the most effective extracts. Kos et al. (2017) reported that the addition of bay leaf essential oil (0.005% and 0.01%) had an unfavorable effect on the sensory properties of dry fermented game sausages. Conversely, da Silveira et al. (2014) stated that incorporating bay leaf essential oil at concentrations of 0.1 and 0.05 g/100 g into fresh Tuscan sausage extended the shelf life of the sausage for 2 days, and the sausages were acceptable at both concentrations in sensory analysis. Thus, the aim of this study was to investigate the quality attributes and biogenic amine contents of the fermented sausages (sucuk) incorporated with bay leaf extract at different levels during production and storage.

## 2 MATERIALS AND METHODS

Bay leaf was harvested from naturally grown shrubs in the district of Cadirli in Mersin, Turkey (36°21′12.0″ N 33°47′58.4″ E). Dried bay leaves were obtained from a local manufacturer. Beef (80% lean meat and 20% fat) was purchased from a local meat processing plant in the district of Havutlu in Adana, Turkey. Sucuks were manufactured in Cukurova University Pilot Sucuk Plant. All chemicals used in the study were analytical grade.

### 2.1 Preparation of bay leaf extract

In a preliminary experiment, five different bay leaf extracts (distilled water and 60%, 70%, 80%, and 90% ethanol extracts) were prepared to determine the extract with the highest antioxidant capacity. Dried bay leaves were ground to obtain a fine powder. Twenty‐five grams of bay leaf powder was weighed in 250 mL of distilled water and left for 24 h with constant shaking at 25°C in a water bath (Memmert, WNB22, Schwabach, Germany) according to Bozkurt (2006). Twenty‐five grams of bay leaf powders were also weighed in 250 mL of 60%, 70%, 80%, and 90% ethanol, respectively. Then the mixtures were left in ultrasound bath (Bandelin Sonorex RK 1028 H, Bandelin Electronic, Berlin, Germany) for 1 h at room temperature (Kurcubic et al., 2014). After filtration through coarse filter papers, all extracts were concentrated in a rotary evaporator (Hei‐VAP Advantage Rotary Evaporator, Heidolph Instruments, Schwabach, Germany) at 40°C under vacuum to obtain approximately 50 mL of each extracts. Production of each bay leaf extract using distilled water and different concentrations of ethanol was replicated three times. Then the extracts were subjected to total phenolic content and antioxidant activity analyses to determine antioxidant capacities.

### 2.2 Total phenolic content

Bay leaf extracts were diluted with 80% methanol (1:10 v/v) and centrifuged (Hettich Zentrifugen Mikro 220R, Andreas Hettich GmbH & Co., Tuttlingen, Germany) at 4000 rpm at 4°C for 10 min. Then, 100 μL of supernatant was transferred into a glass tube and mixed with 100 μL Folin–Ciocalteu solution and 3000 μL distilled water. Following 10 min of incubation, 100 μL of 20% (w/v) Na_2_CO_3_ solution was transferred into the tube. Then, the mixture was kept in dark for 2 h before measuring absorbance at 765 nm using a spectrophotometer (Lambda 25 UV/Vis Spectrometer, Perkin Elmer, CT). A calibration curve was prepared using different concentrations of gallic acid solution to calculate total phenolic contents of the samples. The results were presented as mg gallic acid equivalent/g dry weight (Abdullakasim et al., 2007; Agcam, 2022).

### 2.3 Antioxidant activity

The antioxidant activities of bay leaf extracts were determined by DPPH (1,1‐diphenyl‐2‐picrylhydrazyl) free radical‐scavenging method according to a previously described procedure by Agcam et al. (2021) and Klimczak et al. (2007) with some modifications. Bay leaf extracts were diluted with 80% methanol (1:40 *v/v*) and centrifuged (Hettich Zentrifugen Mikro 220R, Andreas Hettich GmbH & Co., Tuttlingen, Germany) at 4000 rpm at 4°C for 20 min. Then, 100 μL of supernatants were transferred into glass tubes and mixed with 3000 μL DPPH* radical (0.050 g/L in 80% methanol) using vortex. The control was prepared with 100 μL of distilled water. The absorbance of the samples were taken using a spectrophotometer (Lambda 25 UV/Vis Spectrometer, Perkin Elmer, CT) at 515 nm after keeping samples in the dark to equilibrate the reaction for 60 min. Five separate tubes were prepared for each sample. The antioxidant activity of the bay leaf extracts were expressed as μmol Trolox equivalent/g dry weight.

### 2.4 Preparation of fermented sausage

In Cukurova University Pilot Sucuk Plant, five groups of sucuks were manufactured. Three groups contained 1 (E‐1), 5 (E‐5), and 10 mL/kg (E‐10) of bay leaf extract (70% ethanol extract). One group contained 500 mg/kg ascorbic acid (AA), while the control group did not contain any antioxidant. Sausage dough was prepared by grinding (using 3 mm grinder plate) 80% beef and 20% beef fat. Then, 17.5 g of salt, 0.15 g of sodium nitrite, 2 g of sucrose, 10 g of fresh garlic, 7 g of ground red pepper, 5 g of black pepper, 9 g of cumin, 2.5 g of allspice, and starter culture mix (*Staphylococcus carnosus* and *Lactobacillus sakei*) were added for 1 kg of meat base. After an adequate mixing, sausage dough was divided into five equal batches. Bay leaf extracts and AA were added in four randomly selected batches. All batches were mixed and stuffed into natural beef casings with a diameter of 44 mm to obtain approximately 150 g of portions (a total of 75 sucuks). Then, sausages were fermented at 22 ± 1°C and 85%–90% relative humidity for the first day, at 20 ± 1°C and 85%–90% relative humidity for the second day, and at 18 ± 1°C and 80%–85% relative humidity for the third day in a fermentation unit. Following the fermentation, sausages were dried in the same unit at 16°C ± 1 and 70%–75% relative humidity for 3 days. The sucuk samples were than vacuum packaged and stored at 4°C for 60 days. Samples were collected before fermentation, after fermentation, from the end product, at 30 days of storage, and at 60 days of storage. The whole experiment was replicated two times.

### 2.5 pH, titratable acidity, and moisture content

The pH values were measured using a calibrated pH meter (S220, Mettler‐Toledo, LLC, Columbus, OH) in slurries prepared by blending 5 g of samples with 45 mL of distilled water (Ockerman, 1985). Then each sample was titrated using 0.1 N NaOH. The total acidity was calculated as percent lactic acid (Gökalp et al., 2001). The moisture content of the samples were determined using oven (Memmert, Universal Oven Tech., Germany) drying method at 100°C for 18 h (Nielsen, 2003).

### 2.6 Color analysis

Color space values including L* (lightness), a* (redness), and b* (yellowness) were taken from inner surfaces of samples. A Konica Minolta colorimeter (Chroma Meter CR‐400, Konica Minolta Sensing Inc., Japan) was calibrated with a standard white tile (*Y* = 93.7, *x* = 0.3157, *y* = 0.3323). The colorimeter was equipped with an 8‐mm aperture size and was set to illuminant D‐65 and 2° observer. Three measurements were taken from inner surfaces of each sample (Benli & Tokgoz, 2021; Calnan et al., 2016).

### 2.7 Texture profile analysis

A texture analyzer (Model TA–XT Plus, Stabile Microsystems, England) was used to determine texture profile analysis (TPA) values including hardness, adhesiveness, cohesiveness, chewiness, and resilience. Three sausage samples (1 cm height × 3 cm diameter) were compressed in a double compression cycle axially to 75% of their initial height. The texture analyzer was equipped with 50 kg load cell and a 50‐mm cylindrical probe (TA‐25, 2″ dia. cyl., alum., England). Testing parameters were set to 3.0 mm/s pretest speed, 1.0 mm/s test speed, and 3.0 mm/s posttest speed (Benli & Tokgoz, 2021; Kargozari et al., 2014).

### 2.8 Biogenic amine analysis

Determination of biogenic amine contents was performed using the method of Eerola et al. (1993) and Gençcelep et al. (2008) with some modifications. The biogenic amines were extracted from fermented sausages using 0.4 M perchloric acid and their dansyl derivatives were detected by HPLC/PDA. In this respect, 2.0 g of sample was homogenized with 20 mL of 0.4 M perchloric acid solution using an Ultra‐Turrax (T‐18 digital Ultra‐Turrax, IKA Works GmbH & Co. KG, Staufen, Germany) homogenizer (8000 rpm, 60 s). Then, the extract was centrifuged (Hettich Zentrifugen Mikro 220R, Andreas Hettich GmbH & Co., Tuttlingen, Germany) at 3000 rpm for 10 min, filtered through a coarse filter paper, and completed to 50 mL with 0.4 M perchloric acid solution. For the sample derivatization, 1 mL of the extract was made alkaline with 200 μL of 2 N NaOH and 300 μL of saturated NaHCO_3_ was added. Then, 2 mL of dansyl chloride solution (10 mg/mL in acetone, w/v) was added and the mixture was incubated (Memmert, Universal Oven Tech., Germany) for 45 min at 40°C. Next, 100 μL (25%) ammonia was added and the total volume was completed to 5 mL with acetonitrile. Then, the sample was centrifuged (Hettich Zentrifugen Mikro 220R, Andreas Hettich GmbH & Co., Tuttlingen, Germany) at 3000 rpm for 5 min and passed through a 0.45‐μm PTFE filter. To prepare the standard curves, biogenic amine standards were completed to 1 mL with 0.4 M perchloric acid solution and derivatized as in the sample derivatization. Ammonium acetate (0.1 M; solvent A) and acetonitrile (solvent B) were used as mobile phases. The mobile phase gradient elution was 50% A + 50% B at the beginning (0 min), 10% A + 90% B at 25 min, and 50% A + 50% B at 35 min. The flow rate was set to 1 mL/min and the column temperature was kept at 40°C. The injection volume of the sample was 20 μL into the column (Nukleodur 100‐5‐C18; 12.5 × 4 mm). The measurements were performed at 254 nm. HPLC system comprised a HPLC (Shimadzu, Japan), a quaternary pump (LC‐20AT), a vacuum degasser (DGU‐20A5), an auto sampler (SIL‐20A), a column oven (CTO‐10AS), a photodiode array detector (SPD‐M20A), and a computer with Shimadzu LC solution package program. Biogenic amines including tryptamine, 2‐phenylethylamine, putrescine, cadaverine, histamine, tyramine, spermidine, and spermine were determined in this study (Figure S1). The identification of the peaks was conducted by comparing retention time and spectral data of the certified commercial standards. The concentration of the peaks was quantified using calibration curves obtained from five different concentration points of these standards (*R* ^2^ = 0.997–0.999).

### 2.9 Thiobarbituric acid reactive substances

Thiobarbituric acid reactive substances (TBARS) values were measured using the method of Mielnik et al. (2006) with some modification. Five gram sausage was homogenized with 30 mL of 7.5% aqueous solution of trichloroacetic acid using a Ultra‐Turrax (T‐18 digital Ultra‐Turrax, IKA Works GmbH & Co. KG, Staufen, Germany) homogenizer (9500 rpm, 60 s). Then the homogenate was centrifuged (Hettich Zentrifugen Mikro 220R, Andreas Hettich GmbH & Co., Tuttlingen, Germany) at 6000 rpm for 20 min at 20°C. After filtration (Whatman No.1 filter) using vacuum filter, 5.0 mL extract was transferred in a stopper test tube and 5.0 mL of 0.02 M aqueous thiobarbituric acid (TBA) was added. The test tubes were incubated in a water bath at 100°C for 35 min. Then the tubes were cooled under running water. Absorbance measurements were taken at 532 nm (Lambda 25 UV/Vis Spectrometer, Perkin Elmer, CT) against a blank sample prepared with 5 mL distilled water and 5 mL TBA solution. A standard curve was prepared with 1,1,3,3 tetraethoxypropane standards and TBARS values were expressed as mg malondialdehyde/kg of sausage.

### 2.10 Sensory evaluation

Fermented sausage samples were evaluated by 10 panelists (regular sucuk consumers) consisting of faculty members and graduate students from the Department of Food Engineering at Cukurova University. The panelist were informed and given instructions before evaluating samples. They evaluated the cooked sucuks in terms of appearance, color, aroma, taste, texture, and overall liking. Sensory evaluation was replicated by the same panelists in two sessions in morning and afternoon. The panelists were seated to individual booths in the sensory evaluation room and preliminary information was given about the sensory evaluation. Meanwhile, sucuk samples were cut into 1 cm slices in the preparation room and cooked for 1 min on an electric grill (Grill Comfort, Tefal, France). Three‐digit random numbers were used in the coding of the samples. Each sample was given scores using a hedonic scale of 1 (*dislike extremely*) to 9 (*like extremely*). Water and salt‐free biscuits were given to the panelists to avoid interaction between the samples (Arslan & Soyer, 2018; Meilgaard et al., 1999).

### 2.11 Statistical analyses

Statistical analyses of data were performed with SPSS software version 20 (IBM SPSS Statistics, Armonk, NY). One‐way ANOVA procedure was applied in a completely randomized design to determine significant differences among the treatments for physicochemical analyses. After a significant difference (*p* < .05) was determined, Duncan multiple comparison test was used to compare the treatment means (Ott & Longnecker, 2001). Statistical analysis of sensory data was performed using one‐way ANOVA procedure in a randomized complete block design. Five treatment groups were included in the model as a fixed effect, while the panelists were considered as a random effect (Arslan & Soyer, 2018; Meilgaard et al., 1999).

## 3 RESULTS AND DISCUSSION

### 3.1 Total phenolic content and antioxidant activity of bay leaf extracts

**FIGURE 1:**
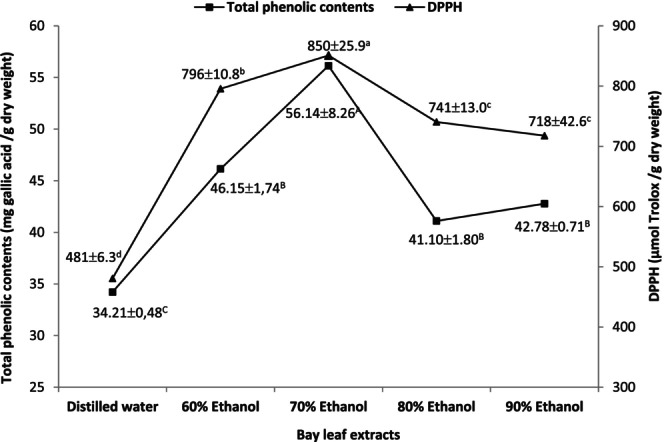
The total phenolic contents and antioxidant activity values (±SD) of five different bay leaf extracts. Different uppercase and lowercase letters are significantly different (*p* < .05) for total phenolic contents and antioxidant activities, respectively.

Figure 1 represents the total phenolic contents and antioxidant activity (DPPH free radical‐scavenging) values of five different bay leaf extracts obtained using distilled water and 60%, 70%, 80%, and 90% ethanol:distilled water mixtures. The highest total phenolic content (56.14 mg gallic acid/g dry weight) and antioxidant activity (850 μmol Trolox/g dry weight) values were determined with 70% ethanol extract (*p* < .05). Conversely, distilled water extract produced the lowest total phenolic content (34.21 mg gallic acid/g dry weight) and antioxidant activity (481 μmol Trolox/g dry weight) values. In recent years, production of natural antioxidants from plant sources has increased due to safety and health concerns related to the use of synthetic antioxidants, including BHT, BHA, *tert*‐butylhydroquinine, and propyl gallate (Altemimi et al., 2017). The solvents are used to extract phytochemicals, as natural antioxidants, from various parts of plants. Ethanol and water are categorized among the green solvents due to nontoxic, biodegradable, recyclable, and renewable nature of these solvents. Although it is the most preferred green solvent, water could extract all hydrophilic substances, including phenols, saponins, and polysaccharides, in consequence of its nonselective nature. Conversely, ethanol was reported to be selective in action and mostly used to extract polyphenols, while the efficiency of ethanol extraction could be further modified by mixing ethanol with water (Kumar et al., 2023; Muniz‐Marquez et al., 2013). In addition, there was a positive correlation (*r* = .887, *p* < .05) between the total phenolic contents and antioxidant activities of five different bay leaf extracts in this study. Similarly, a positive correlation was reported between total phenolic contents and antioxidant activities in various plant extracts (Altemimi et al., 2017; Javanmardi, 2003; Wong & Kitts, 2006). Furthermore, ultrasound‐assisted extraction was reported to enhance the extraction of phenolic compounds from bay leaf due to the disruption effects of cavitation bubbles on biological cell walls (Muniz‐Marquez et al., 2013). Thus, ultrasound‐assisted 70% ethanol extraction of bay leaf was selected as the most suitable method to obtain a bay leaf extract for the incorporation with fermented sausage (sucuk) in this study.

### 3.2 pH and titratable acidity values

**FIGURE 2:**
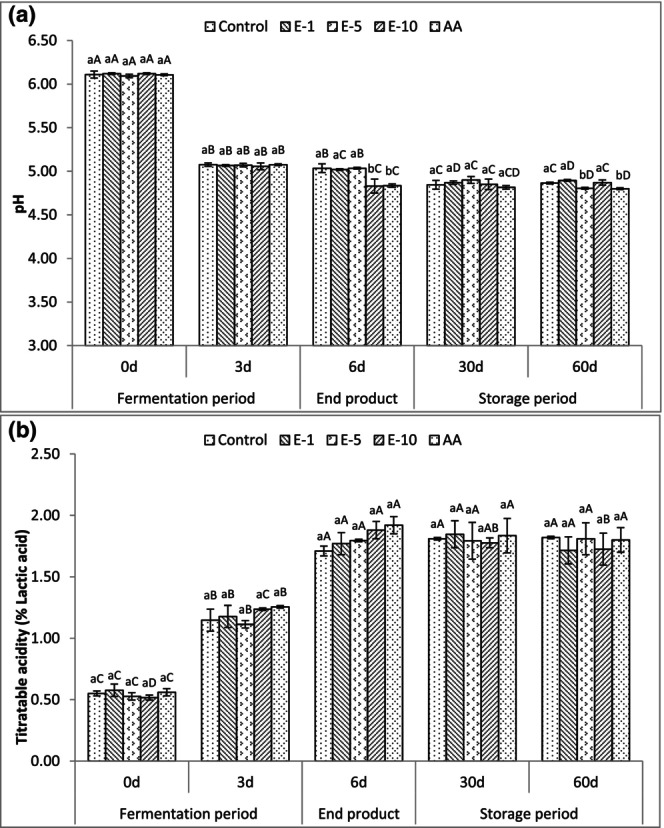
Mean pH values (a) and titratable acidity (b) of fermented sausages (sucuk) during production and storage. Bars represent standard deviations (SD). The difference between the values indicated with different lowercase letters on the same day is significant (*p* < .05). The difference between the values indicated with different uppercase letters for each treatment group is significant (*p* < .05) during production and storage. Control: control group; E‐1: 1 mL/kg bay leaf extract added group; E‐5: 5 mL/kg bay leaf extract added group; E‐10: 10 mL/kg bay leaf extract added group; and AA: 500 mg/kg ascorbic acid added group.

The pH and titratable acidity values of fermented sausages during production and storage are presented in Figure 2. Predictably, pH values decreased and titratable acidity values increased significantly (*p* < .05) for all sucuks from day 0 to day 3 during the fermentation. The initial average pH values of sucuks were between 6.09 and 6.12, while pH values were between 5.06 and 5.08 on day 3. None of the treatments produced a significantly different pH and titratable acidity values during the fermentation. However, the effect of the treatments on the pH values of sucuks was significant (*p* < .05) for the end product. The control (5.04), E‐1 (5.02), and E‐5 (5.04) had the highest pH values, while E‐10 (4.83) and AA (4.84) had the lowest pH values for the end products. Similarly, numerically higher titratable acidity values were observed on E‐10 and AA groups on the end product even though the effect of the treatments was not significant. Moreover, there were decreases on pH values of all treatments during storage (*p* < .05) with the exception of E‐10. The pH values ranged from 4.82 to 4.90 and 4.80 to 4.90 on day 30 and day 60, respectively. In addition, there were no differences on titratable acidity values for all treatments during storage except for E‐10 (*p* < .05), which had titratable acidity values of 1.88% and 1.73% on the end product and day 60, respectively. Overall, the fluctuation in pH and titratable acidity values occurred in a limited range following the fermentation period. The decrease in pH and increase in titratable acidity values during the fermentation was related to the conversion of carbohydrates by lactic acid bacteria and the accumulation of lactic acid in the fermented sausages (Bover‐Cid et al., 2001). According to Demeyer and Stahnke (2002), after the acidity caused by fermentation in fermented sausage products, the solubilized muscle proteins coagulate and form a gel around fat and meat particles. The hardness of the sausage begins to rise at a pH of 5.4 and continues to rise progressively until the pH drops to 4.9. Although most of the studies in the literature indicated a high level of variability among the pH values (4.53–6.74) of sucuk samples collected from the Turkish market (Benli, 2017; Benli & Barutcu, 2021; Erkmen & Bozkurt, 2004; Gençcelep et al., 2008), in this study, pH decreases were in the expected ranges for manufacturing a traditional fermented sucuk, and the incorporation of bay leaf extract produced similar pH values when compared to control and AA samples.

### 3.3 Moisture contents

**FIGURE 3:**
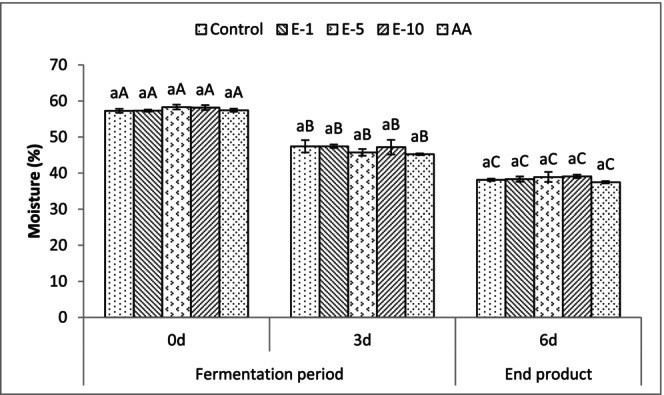
Mean moisture values of fermented sausages (sucuk) during production. Bars represent standard deviations (SD). The difference between the values indicated with different lowercase letters on the same day is significant (*p* < .05). The difference between the values indicated with different uppercase letters for each treatment group is significant (*p* < .05) during production and storage. Control: control group; E‐1: 1 mL/kg bay leaf extract added group; E‐5: 5 mL/kg bay leaf extract added group; E‐10: 10 mL/kg bay leaf extract added group; and AA: 500 mg/kg ascorbic acid added group.

The moisture contents of fermented sausages during production are presented in Figure 3. Moisture contents of all treatments decreased (*p* < .05) during the production of sucuks. Initial average moisture contents ranged from 57.28% to 58.31%; following the fermentation and drying periods the end products had average moisture contents ranging from 37.48% to 39.09%. None of the treatments had a significant effect on the moisture contents of sucuks during production, indicating that the addition of bay leaf extracts produced similar moisture contents to the control and AA groups. The previous studies (Benli & Barutcu, 2021; Con & Gökalp, 1998; Siriken et al., 2009) indicated that sucuk samples collected from the different regions in Turkey had highly variable moisture contents (20.96%–50.49%). Nonetheless, in this study, variation of the moisture contents in sucuk samples was in the expected ranges for manufacturing a traditional fermented sucuk (moisture content of <40%) even after the incorporation of bay leaf extracts when compared to the control and AA groups, which indicated a lack of standardized manufacturing methods for sucuks in the Turkish market.

### 3.4 Color values

The color values (L*, a*, b*) of fermented sausages during storage are presented in Table 1. The L* values decreased statistically only in E‐10 samples during storage (*p* < .05). The L* values varied from 50.83 to 53.84 in the end products, whereas they varied from 51.18 to 53.22 on day 60. Thus, variations in L* values were numerically constrained during storage for all treatments. The a* values decreased in E‐1, E‐5, and AA samples, while the b* values decreased in control, E‐5, E‐10, and AA samples during the storage (*p* < .05). The initial a* and b* values of sucuks varied from 12.80 to 13.45 and from 11.48 to 13.59 in the end products, respectively, whereas they varied from 11.27 to 11.95 and from 10.23 to 11.93 on day 60, indicating decreases in the redness and yellowness of these samples over time. In addition, effects of the treatments on L*, a*, and b* values were not significant except for b* values on day 60, which were also in a numerically limited range. Before making a purchase, consumers are influenced by the expected product quality of food products. Consumers look for quality indicators while purchasing food items to predict their quality performance when consuming them. One of the most crucial quality cues for consumer acceptance of meat products is color (Becker, 2002). Search attributes, experience attributes, and credence attributes are the three main categories of quality attributes that have been established in the literature. Consumers make a buying decision among many alternatives to foods while purchasing using search attributes, commonly referred to as quality cues. The two different categories of quality cues are intrinsic and extrinsic cues. Extrinsic cues refer to information that is not directly related to the physical properties of the product, whereas intrinsic cues refer to the inherent visible aspects of a product. Consumers form quality expectations using intrinsic and extrinsic cues to determine the eating quality of a product while consuming. Color, marbling, and fat content were reported as the most important intrinsic cues for beef, whereas origin and place of purchase were indicated as the most important extrinsic cues (Becker, 2002; Henchion et al., 2014). The unique color of meat products is mostly attributed to the use of nitrite and/or nitrate during the curing process. Curing is one of the essential processing steps, not only for the color development but also to give a unique flavor and texture and to prevent microbial growth (Benli, 2019). In this study, the results of the color values indicated that the incorporation of bay leaf extracts produced color values that were comparable to the control and AA groups even though indications of a decrease were observed during storage of sucuks in some treatments for a* (E‐1, E‐5, and AA) and b* (control, E‐5, E‐10, and AA) values. Thus, the incorporation of bay leaf extracts might not affect the consumer acceptability of sucuks due to the color variations as an intrinsic cue.

**TABLE 1:** Mean color values of fermented sausages (sucuk) during storage.

Color	Storage	Control	E‐1	E‐5	E‐10	AA
Lightness (L*)	End product	52.57 ± 0.33^aA^	52.10 ± 0.74^aA^	53.19 ± 1.60^aA^	53.84 ± 0.78^aA^	50.83 ± 2.06^aA^
	30 days	51.62 ± 0.14^aA^	52.10 ± 0.86^aA^	51.50 ± 1.23^aA^	52.16 ± 0.33^aB^	51.77 ± 1.61^aA^
	60 days	52.78 ± 1.82^aA^	53.22 ± 1.20^aA^	53.22 ± 0.23^aA^	51.18 ± 0.62^aB^	51.38 ± 0.13^aA^
Redness (a*)	End product	13.43 ± 0.37^aA^	13.27 ± 0.64^aA^	13.07 ± 0.01^aA^	13.45 ± 1.19^aA^	12.80 ± 0.01^aA^
	30 days	12.23 ± 0.15^aA^	12.00 ± 0.32^aB^	11.95 ± 0.23^aB^	11.76 ± 0.40^aA^	11.95 ± 0.34^aB^
	60 days	11.80 ± 0.78^aA^	11.95 ± 0.09^aB^	11.75 ± 0.06^aB^	11.27 ± 0.32^aA^	11.65 ± 0.12^aB^
Yellowness (b*)	End product	13.08 ± 0.16^aA^	12.45 ± 1.12^aA^	12.97 ± 0.24^aA^	13.59 ± 1.09^aA^	11.48 ± 0.01^aA^
	30 days	10.89 ± 0.01^aB^	11.17 ± 0.48^aA^	11.14 ± 0.44^aB^	11.47 ± 0.37^aB^	11.15 ± 0.04^aB^
	60 days	11.43 ± 0.40^abB^	11.93 ± 0.25^aA^	10.78 ± 0.47^bcB^	10.23 ± 0.68^cB^	10.49 ± 0.03^bcC^

*Note*: The difference between the mean values (±SD) indicated with different lowercase letters on the same row is significant (*p* < .05). The difference between the mean values (±SD) indicated with different uppercase letters on the same column is significant (*p* < .05) for each color value. Control: control group; E‐1: 1 mL/kg bay leaf extract added group; E‐5: 5 mL/kg bay leaf extract added group; E‐10: 10 mL/kg bay leaf extract added group; and AA: 500 mg/kg ascorbic acid added group.

### 3.5 Texture profile analysis

The TPA values of fermented sausages during storage are presented in Table 2. The texture of meat products is a key component of their organoleptic properties and has an immediate effect on consumers since the texture is one of the main variables associated to quality attributes of the meat products (Chorbadzhiev et al., 2017). In this study, effect of the treatments was not significant at any storage period. However, the storage had significant effects on hardness, adhesiveness, and chewiness values in several treatments (*p* < .05). The hardness values increased in E‐1, E‐5, and E‐10, the adhesiveness values increased in E‐1 and E‐10, and the chewiness values increased in control and E‐10 treatments during storage. As a TPA component, hardness was defined as the maximum force necessary to compress a sample during the first cycle of compression. The work necessary for overcoming the attractive forces between the sample surface and another contacted surface was referred to as adhesiveness. Furthermore, the amount of chewing required masticating the sample before swallowing is called chewiness (Benli, 2016; Petracci et al., 2014; Yuca et al., 2019). Although there was an indication that the incorporation of bay leaf extracts might cause increases in the hardness, adhesiveness, and chewiness values of sucuk, there were no significant differences among the treatments at any storage period. Furthermore, neither storage nor treatments had a significant effect on cohesiveness and resilience values of samples. In general, the main textural changes in a fermented sausage occur mostly during fermentation due to decrease in pH and coagulation of the solubilized muscle proteins to form a gel around fat and meat particles (Li et al., 2023). Thus, the results indicated that the incorporation of bay leaf extracts might result in TPA values that were similar to the control and AA groups.

**TABLE 2:** Mean texture profile analysis (TPA) values of fermented sausages (sucuk) during storage.

TPA	Storage	Control	E‐1	E‐5	E‐10	AA
Hardness (N)	End product	132.66 ± 18.73^aA^	146.44 ± 4.66^aB^	127.36 ± 5.34^aB^	118.50 ± 3.91^aB^	124.40 ± 6.01^aA^
	30 days	142.32 ± 5.65^aA^	150.58 ± 6.20^aB^	138.62 ± 8.42^aAB^	132.59 ± 9.54^aB^	129.58 ± 12.07^aA^
	60 days	163.29 ± 8.32^aA^	168.23 ± 0.36^aA^	154.57 ± 6.17^aA^	154.05 ± 7.43^aA^	153.46 ± 12.62^aA^
Adhesiveness (N.s)	End product	−297.3 ± 48.3^aA^	−302.7 ± 1.9^aB^	−262.7 ± 23.7^aA^	−256.8 ± 5.4^aB^	−247.7 ± 28.6^aA^
	30 days	−323.3 ± 27.3^aA^	−363.24 ± 30.0^aAB^	−305.4 ± 40.7^aA^	−276.3 ± 46.1^aB^	−261.8 ± 58.3^aA^
	60 days	−401.5 ± 32.8^aA^	−416.3 ± 31.0^aA^	−391.7 ± 38.3^aA^	−418.1 ± 22.2^aA^	−333.8 ± 26.7^aA^
Cohesiveness	End product	0.466 ± 0.011^aA^	0.453 ± 0.005^aA^	0.475 ± 0.025^aA^	0.474 ± 0.023^aA^	0.485 ± 0.007^aA^
	30 days	0.459 ± 0.006^aA^	0.451 ± 0.006^aA^	0.463 ± 0.008^aA^	0.469 ± 0.010^aA^	0.473 ± 0.012^aA^
	60 days	0.433 ± 0.011^aA^	0.444 ± 0.012^aA^	0.429 ± 0.012^aA^	0.450 ± 0.025^aA^	0.463 ± 0.004^aA^
Chewiness (N)	End product	37.27 ± 1.29^aB^	39.23 ± 6.23^aA^	35.42 ± 0.54^aA^	31.45 ± 2.56^aB^	36.76 ± 1.32^aA^
	30 days	38.42 ± 1.06^aB^	39.96 ± 1.16^aA^	37.73 ± 1.58^aA^	36.60 ± 1.78^aA^	36.04 ± 2.26^aA^
	60 days	44.00 ± 1.93^aA^	41.33 ± 0.25^aA^	36.70 ± 2.59^aA^	40.70 ± 0.51^aA^	44.42 ± 7.54^aA^
Resilience	End product	0.133 ± 0.013^aA^	0.134 ± 0.001^aA^	0.139 ± 0.008^aA^	0.133 ± 0.006^aA^	0.139 ± 0.001^aA^
	30 days	0.135 ± 0.001^aA^	0.134 ± 0.001^aA^	0.136 ± 0.001^aA^	0.136 ± 0.001^aA^	0.137 ± 0.001^aA^
	60 days	0.131 ± 0.003^aA^	0.138 ± 0.006^aA^	0.128 ± 0.001^aA^	0.133 ± 0.005^aA^	0.139 ± 0.004^aA^

*Note*: The difference between the mean values (±SD) indicated with different lowercase letters on the same row is significant (*p* < .05). The difference between the mean values (±SD) indicated with different uppercase letters on the same column is significant (*p* < .05) for each physical property. Control: control group; E‐1: 1 mL/kg bay leaf extract added group; E‐5: 5 mL/kg bay leaf extract added group; E‐10: 10 mL/kg bay leaf extract added group; and AA: 500 mg/kg ascorbic acid added group.

### 3.6 Biogenic amines

The biogenic amine values of fermented sausages during production and storage are presented in Table 3. Biogenic amines can be expected in almost all foods that include proteins or free amino acids and are exposed to conditions that support microbial or biochemical activities. However, the types of food and microorganisms present have a significant impact on the overall quantity of the various biogenic amines produced (Silla Santos, 1996). In this study, the effect of the treatments on tryptamine values of sucuk samples was significant for each production and storage period (*p* < .05) except for day 3 (Table 3). Furthermore, changes in tryptamine contents of each treatment were significant during production and storage (*p* < .05). The highest tryptamine values were determined on day 0 for all treatments ranging from 19.44 to 30.66 mg/kg dry weight. Although the tryptamine contents continued to decrease during production and on day 30 of storage, there were increases in tryptamine levels on day 60 of storage except for E‐10. The tryptamine content of E‐10 decreased on day 3 of production and remained similar in the end product and during storage. The tryptamine levels of all treatments were between 10.18 and 15.58 mg/kg dry weight at the end of storage. Erkmen and Bozkurt (2004) indicated that tryptamine was one of the most abundant biogenic amines detected in 50 sucuk samples collected from factories and butchers and the use of starter cultures and lower pH values in factory sucuks were associated with the lower levels of tryptamine. In this study, the use of starter cultures and lower pH values could be associated with the lower levels and limited fluctuation of tryptamine values observed among all treatments during manufacturing and storage. Furthermore, decreases in tryptamine values could be attributed to microbial degradation in which tryptamine was used as a nitrogen source (Erkmen & Bozkurt, 2004; Wang, Zhang, et al., 2022).

**TABLE 3:** Mean biogenic amine values of fermented sausages (sucuk) during production and storage.

Biogenic amines (mg/kg dry weight)	Storage	Control	E‐1	E‐5	E‐10	AA
Tryptamine	0 day	22.53 ± 0.66^bcA^	21.53 ± 0.58^bcA^	25.51 ± 3.71^bA^	30.66 ± 1.53^aA^	19.44 ± 0.06^cA^
	3 days	10.86 ± 1.13^aBC^	10.76 ± 1.35^aB^	12.03 ± 0.19^aBC^	10.28 ± 0.27^aB^	9.03 ± 0.22^aC^
	End product	10.19 ± 0.04^aBC^	6.97 ± 0.54^bcC^	8.68 ± 0.63^abCD^	8.42 ± 0.99^abB^	6.56 ± 0.82^cD^
	30 days	8.24 ± 0.35^aC^	7.25 ± 0.45^bcC^	6.96 ± 0.31^cD^	8.08 ± 0.39^abB^	6.43 ± 0.09^cD^
	60 days	11.39 ± 1.86^cB^	12.54 ± 0.33^bcB^	15.58 ± 0.29^aB^	10.18 ± 0.50^cB^	14.80 ± 1.12^abB^
2‐Phenylethylamine	0 day	32.04 ± 7.75^aA^	25.71 ± 1.39^aA^	31.39 ± 3.69^aA^	27.46 ± 1.02^aA^	29.13 ± 2.68^aA^
	3 days	3.47 ± 0.18^aB^	3.90 ± 2.01^aB^	2.95 ± 0.32^aB^	2.39 ± 0.54^aC^	2.56 ± 0.07^aB^
	End product	2.85 ± 0.43^bcB^	4.27 ± 0.50^abB^	2.24 ± 0.01^cB^	3.56 ± 1.01^abcC^	4.79 ± 0.42^aB^
	30 days	6.18 ± 2.31^aB^	5.25 ± 1.64^aB^	5.09 ± 0.38^aB^	7.18 ± 2.84^aBC^	6.41 ± 2.04^aB^
	60 days	8.95 ± 0.85^aB^	5.18 ± 1.13^aB^	4.52 ± 1.24^aB^	8.41 ± 2.46^aB^	11.00 ± 6.96^aB^
Putrescine	0 day	7.13 ± 0.51^aA^	6.32 ± 0.56^aA^	7.98 ± 0.85^aA^	7.43 ± 2.17^aA^	6.86 ± 0.65^aA^
	3 days	3.29 ± 0.22^aB^	2.44 ± 1.06^aBC^	3.51 ± 0.94^aB^	2.93 ± 1.05^aC^	4.20 ± 0.49^aA^
	End product	3.31 ± 0.36^cB^	5.85 ± 0.09^aA^	3.11 ± 0.64^cB^	4.62 ± 0.45^bABC^	5.60 ± 0.27^aA^
	30 days	3.06 ± 0.09^bB^	4.35 ± 1.13^bAB^	3.46 ± 0.52^bB^	6.33 ± 0.37^aAB^	7.05 ± 0.97^aA^
	60 days	2.77 ± 0.25^aB^	2.19 ± 0.44^aC^	3.22 ± 0.45^aB^	3.25 ± 0.96^aBC^	4.62 ± 2.03^aA^
Cadaverine	0 day	10.75 ± 1.11^aA^	8.96 ± 0.34^aA^	10.72 ± 1.07^aA^	12.21 ± 2.22^aA^	11.93 ± 0.70^aA^
	3 days	5.29 ± 0.33^aC^	5.38 ± 0.65^aB^	4.38 ± 0.93^aB^	4.72 ± 0.69^aB^	5.42 ± 0.20^aB^
	End product	3.50 ± 0.05^bD^	4.58 ± 0.40^aB^	3.28 ± 0.23^bB^	4.10 ± 0.56^abB^	4.73 ± 0.03^aB^
	30 days	3.68 ± 0.04^aD^	3.37 ± 0.32^aC^	4.28 ± 0.53^aB^	4.34 ± 0.16^aB^	4.37 ± 0.15^aB^
	60 days	7.17 ± 0.63^aB^	4.91 ± 0.20^bB^	4.71 ± 0.09^bB^	4.80 ± 0.56^bB^	5.84 ± 0.98^abB^
Histamine	0 day	10.80 ± 2.30^aA^	8.76 ± 0.50^aA^	9.94 ± 1.55^aA^	11.38 ± 1.86^aA^	9.06 ± 0.21^aA^
	3 days	8.24 ± 0.39^aA^	8.30 ± 0.45^aA^	7.81 ± 0.28^aA^	8.64 ± 0.21^aA^	7.97 ± 0.43^aB^
	End product	7.98 ± 0.44^aA^	7.65 ± 0.04^aA^	7.74 ± 0.31^aA^	8.36 ± 0.40^aA^	7.93 ± 0.14^aB^
	30 days	7.63 ± 0.14^bA^	7.99 ± 0.34^bA^	8.96 ± 0.01^aA^	9.40 ± 0.01^aA^	8.81 ± 0.35^aAB^
	60 days	7.79 ± 1.16^aA^	7.59 ± 1.12^aA^	8.92 ± 0.44^aA^	8.68 ± 0.21^aA^	7.01 ± 0.46^aC^
Tyramine	0 day	5.29 ± 1.42^aD^	4.65 ± 0.81^aD^	4.16 ± 0.75^aD^	3.27 ± 0.10^aC^	4.34 ± 0.27^aE^
	3 days	25.61 ± 1.77^aC^	28.67 ± 4.94^aC^	24.19 ± 10.36^aC^	21.11 ± 0.85^aB^	23.87 ± 1.42^aD^
	End product	31.82 ± 4.45^aC^	28.26 ± 0.34^aC^	31.81 ± 5.86^aC^	30.36 ± 6.84^aB^	33.38 ± 0.86^aC^
	30 days	56.24 ± 2.44^aB^	56.81 ± 3.35^aB^	55.27 ± 0.61^aB^	59.32 ± 2.20^aA^	56.54 ± 3.70^aB^
	60 days	68.24 ± 2.77^aA^	65.98 ± 0.71^aA^	71.07 ± 1.05^aA^	61.01 ± 8.64^aA^	65.13 ± 1.95^aA^
Spermidine	0 day	8.01 ± 1.71^aA^	6.06 ± 0.45^aA^	6.58 ± 1.16^aA^	5.66 ± 1.08_aA_	6.30 ± 0.74^aA^
	3 days	6.12 ± 2.35^aA^	4.87 ± 0.02^aA^	5.81 ± 1.63^aA^	6.07 ± 1.24^aA^	5.27 ± 0.57^aA^
	End product	4.94 ± 0.72^aA^	5.65 ± 0.03^aA^	5.26 ± 0.20^aA^	5.29 ± 0.02^aA^	5.64 ± 0.48^aA^
	30 days	5.85 ± 0.04^aA^	5.43 ± 0.80^aA^	5.14 ± 0.21^aA^	5.09 ± 0.33^aA^	5.13 ± 0.15^aA^
	60 days	5.61 ± 0.36^aA^	4.88 ± 0.11^aA^	5.14 ± 0.90^aA^	5.46 ± 0.69^aA^	4.44 ± 0.13^aA^
Spermine	0 day	68.25 ± 14.46^aA^	55.15 ± 0.26^aA^	57.39 ± 2.63^aA^	58.99 ± 2.14^aBC^	54.93 ± 2.96^aA^
	3 days	57.20 ± 1.37^aA^	53.96 ± 0.72^aA^	56.18 ± 0.61^aA^	57.64 ± 1.48^aC^	55.73 ± 0.42^aA^
	End product	58.50 ± 0.28^aA^	56.42 ± 10.63^aA^	56.11 ± 2.04^aA^	56.82 ± 1.60^aC^	56.77 ± 1.79^aA^
	30 days	54.58 ± 1.24^bA^	54.32 ± 2.44^bA^	61.43 ± 1.13^aA^	63.49 ± 0.21^aAB^	61.99 ± 2.33^aA^
	60 days	63.17 ± 4.74^aA^	61.76 ± 1.59^aA^	63.91 ± 3.49^aA^	66.34 ± 2.58^aA^	55.88 ± 2.28^aA^

*Note*: The difference between the mean values (±SD) indicated with different lowercase letters on the same row is significant (*p* < .05). The difference between the mean values (±SD) indicated with different uppercase letters on the same column is significant (*p* < .05) for each biogenic amine. Control: control group; E‐1: 1 mL/kg bay leaf extract added group; E‐5: 5 mL/kg bay leaf extract added group; E‐10: 10 mL/kg bay leaf extract added group; and AA: 500 mg/kg ascorbic acid added group.

The effect of the treatments on 2‐phenylethylamine values of sucuk samples was significant (*p* < .05) only for the end product (Table 3). Thus, the 2‐phenylethylamine values were similar on days 0 and 3 of production, and days 30 and 60 of storage for all treatments. However, changes in 2‐phenylethylamine contents of each treatment were significant during production and storage (*p* < .05). The highest 2‐phenylethylamine values were determined on day 0 for all treatments ranging from 25.71 to 32.04 mg/kg dry weight. With the exception of E‐10 samples, the 2‐phenylethylamine contents of all samples decreased on day 3 of the production and stayed similar in the end product and during storage. The 2‐phenylethylamine content of E‐10 samples decreased during production and increased on day 30 and day 60 of storage. However, the 2‐phenylethylamine contents of all treatments were similar at each storage period and they ranged between 4.52 and 11.00 mg/kg dry weight at the end of the storage. Among the lactic acid bacteria, mostly species from genus *Enterococcus* were reported to decarboxylate phenylalanine to produce 2‐phenylethylamine (Bargossi et al., 2015). Similar to this study, Suvajdzic et al. (2020) found phenylethylamine levels of 25.05 mg/kg in the stuffing of Serbian fermented sausages and they indicated an increase at the ripening stage in phenylethylamine content followed by a decline with the accumulation of tyramine content. The presence of phenylethylamine in raw materials was associated with nonspecific tyrosine decarboxylase activity taking place simultaneously with accumulation of tyramine. In addition, the variety of biogenic amines in raw materials could be affected by the temperature of the environment to which the carcasses were exposed and the microbial flora present on carcass surfaces (Roseiro et al., 2006).

The effect of the treatments on putrescine values of the sucuk samples was significant (*p* < .05) for end product and day 30 samples (Table 3). Thus, the putrescine values were similar on days 0 and 3 of production and day 60 of storage for all treatments. However, changes in putrescine contents of each treatment were significant during production and storage (*p* < .05) except for the AA samples. With the exception of AA treatment, the highest putrescine values for the treatments were determined on day 0 ranging from 6.32 to 7.98 mg/kg dry weight. Putrescine values in the control and E‐5 samples decreased on day 3 of production, and these values did not differ in the end product and during storage (day 30 and day 60). Likewise, putrescine values decreased on day 3 of production in E‐1 and E‐10, however, partial increases were observed in the end product and on day 30 of storage; then, the putrescine values decreased again, resulting in values similar to those on day 3 of production. The putrescine levels of all treatments were between 2.19 and 4.62 mg/kg dry weight at the end of storage. Although some lactic acid bacteria with ornithine decarboxylase activity could produce putrescine, it was suggested that putrescine can be synthesized under circumstances that are unfavorable for enterobacteria when the decarboxylation process plays an important role in bacterial metabolism to produce energy (Miguelez‐Arrizado et al., 2006; Suvajdzic et al., 2020). The putrescine values found in this study were in the lower ranges reported for sucuks (0–255.625 mg/kg), despite the fact that the highest putrescine readings on day 0 indicated poorer hygienic quality with the raw materials (Ekici & Omer, 2018; Roseiro et al., 2006). Furthermore, Van Ba et al. (2016) reported that reducing pH values using starter cultures could decrease putrescine formation, indicating a positive correlation between putrescine content and pH values for fermented sausages. This might explain decreases observed in putrescine values in this study following the fermentation.

The effect of the treatments on the cadaverine values of the sucuk samples was significant (*p* < .05) for the end product and day 60 samples (Table 3). Thus, the cadaverine values were similar on days 0 and 3 of production and day 30 of storage for all treatments. However, changes in cadaverine contents of each treatment were significant during production and storage (*p* < .05). The highest cadaverine values were determined on day 0 for all treatments ranging from 8.96 to 12.21 mg/kg dry weight. Cadaverine values in E‐5, E‐10, and AA treatments decreased on day 3 of production, and these values did not differ in the end product and during storage (day 30 and day 60). Likewise, cadaverine values decreased on day 3 of production in E‐1 and control treatments, however further decreases were observed in the end product and on day 30 of storage; then the cadaverine values increased again on day 60 of storage. The cadaverine levels of all treatments ranged between 4.71 and 7.17 mg/kg dry weight at the end of storage. Putrescine and cadaverine were commonly associated with the hygienic quality of red meat (Wang et al., 2021). In this study, the cadaverine values determined for all treatments were in the lower ranges reported for sucuks (0–1148.75 mg/kg) (Ekici & Omer, 2018). In addition, use of a mixed starter cultures (*Lactobacillus sakei*, *Staphylococcus xylosus*, *Staphylococcus carnosus*) was reported to inhibit Enterobacteriaceae counts and cadaverine levels in fermented mutton sausages (Wang et al., 2021). This could account for decreases in cadaverine levels observed in this study after fermentation. Furthermore, the lowest cadaverine values were determined in E‐1, E‐5, and E‐10 treatments at the end of storage indicating that the incorporation of bay leaf extract could be effective in decreasing cadaverine levels during storage.

The effect of the treatments on histamine values of the sucuk samples was significant (*p* < .05) only for day 30 samples (Table 3). Thus, the histamine values were similar on days 0 and 3 of production, in the end product, and on day 60 of storage for all treatments. The histamine values varied between 8.76 and 11.38 mg/kg dry weight on day 0 for all treatments. Furthermore, changes in histamine contents were significant only for the AA treatment during production and storage (*p* < .05). Histamine values of AA treatment decreased on day 3 of production, end product, and day 30 of storage and had similar values. Even though the AA samples continued to decline on day 60 of storage, there were no significant differences between any of the treatments at the end of the storage ranging from 7.01 to 8.92 mg/kg dry weight. Legal regulations were only established for histamine among the biogenic amines due to the histamine poisoning of humans related to the consumption of fish containing high levels of histamine. The Codex Alimentarius, the European Union, Australia, and New Zealand limited the histamine contents in raw fish to 200 mg/kg, while the United States Food and Drug Administration set an allowable limit of histamine at 50 mg/kg (FAO/WHO, 2018; Visciano et al., 2020). Likewise, histamine content of 100 mg/kg was considered as a reference limit for other products including fermented sausages (Miguelez‐Arrizado et al., 2006). The histamine levels in Turkish fermented sausages were reported ranging from 0 to 469.375 mg/kg (Ekici & Omer, 2018). In this study, histamine contents of all sucuks during production and storage were lower than established reference limits and in the lower ranges reported for sucuks. Furthermore, implementation of good hygienic practices and hazard analysis and critical control point systems were suggested to achieve histamine contents of less than 15 mg/kg in fish (FAO/WHO, 2018). Although numerically higher histamine levels on day 0 indicated a poorer hygienic quality of the raw materials in this study, the little variation in histamine levels across all treatments indicated appropriate hygienic conditions during production and storage.

The effect of the treatments on tyramine values of the sucuk samples was not significant on any of the production and storage periods (Table 3). Thus, the tyramine values were similar on days 0 and 3 of production, in the end product, and on days 30 and 60 of storage for all treatments. However, changes in tyramine contents of each treatment were significant during production and storage (*p* < .05). The lowest tyramine values were determined on day 0 for all treatments ranging from 3.27 to 5.29 mg/kg dry weight. Although tyramine values for all treatments increased during production and storage, there were no differences among the treatments on each production and storage periods and tyramine levels ranged between 61.01 and 71.07 mg/kg dry weight at the end of storage. The most prevalent biogenic amine found in fermented sausages was reported to be tyramine since numerous lactic acid bacteria have the capacity to decarboxylate tyrosine (Miguelez‐Arrizado et al., 2006; Suvajdzic et al., 2020). Similar to the current study, Komprda et al. (2004) observed increase in tyramine levels during the storage of fermented sausages in a study related to influence of spice mixture, the type of starter culture, and storage on biogenic amine content of dry fermented sausages. Contrary, Bozkurt and Erkmen (2004) reported an increase during 15 days of ripening and a decrease during 60 days of storage in the tyramine levels of sucuks. The tyramine levels in Turkish fermented sausages were reported ranging from 0 to 438.125 mg/kg (Ekici & Omer, 2018). Since tyramine exposure of 600 mg per meal was observed to cause no adverse effects in healthy persons (Suvajdzic et al., 2020), the tyramine levels in all sucuks studied in this study were lower than indicated reference limits and within the lower ranges (0–438.125 mg/kg) typically associated with sucuks (Ekici & Omer, 2018) during production and storage.

Spermidine and spermine were considered naturally occurring biogenic amines and already present in raw material. Their contents were not expected to change or vary slightly during production and storage (Bover‐Cid et al., 2001; Komprda et al., 2004; Roseiro et al., 2006). In this study, the effect of the treatments on spermidine values of the sucuk samples was not significant on any of the production and storage periods (Table 3). As a result, spermidine levels of the treatments were comparable on days 0 and 3 of production, in the end product, and on days 30 and 60 of storage. In addition, changes in spermidine contents of each treatment were not significant during production and storage. Furthermore, the effect of the treatments on spermine values of the sucuk samples was significant only for day 30 samples (*p* < .05) and the changes in spermine contents were significant (*p* < .05) only for E‐10 treatment during production and storage (Table 3). However, there were no significant differences between any of the treatments in the end product and at end of the storage. The spermidine and spermine levels (4.44–8.01 mg/kg dry weight and 53.96–68.25 mg/kg dry weight, respectively) in all sucuks studied in this study were in the expected ranges and within the lower ranges (0–554.375 and 0–614.375 mg/kg, respectively) typically associated with sucuks (Ekici & Omer, 2018) during production and storage.

### 3.7 TBARS values

TBARS values of fermented sausages during production and storage are presented in Table 4. The effect of the treatments on TBARS values was significant for days 0 and 3 of production and day 30 of storage (*p* < .05). Thus, the TBARS values were similar on the end product and day 60 of storage for all treatments. However, the changes in the TBARS contents of each treatment were significant during production and storage (*p* < .05). The lowest TBARS values were determined on day 0 for E‐5 (0.35 mg malondialdehyde/kg) and E‐10 (0.32 mg malondialdehyde/kg). The TBARS contents of all samples increased on day 3 of production and decreased in the end product. Although the TBARS contents of the end products were between 0.71 and 1.30 mg malondialdehyde/kg, there were no statistical differences between treatments. The TBARS values were similar on the end product and day 30 of storage for control, E‐1, and E‐5, while it increased for E‐10 and AA samples. Even though E‐10 stayed similar to previous month and the AA sample declined on day 60 of storage, there were no significant differences between any of the treatments at the end of the storage ranging from 1.59 to 1.73 mg malondialdehyde/kg. In traditional Turkish dry fermented sausage (sucuk), TBARS values were reported to decrease with the use of starter culture and increase with the progress of ripening time (Akkose et al., 2023). Ozer and Kilic (2020) indicated that TBARS values increased gradually after fermentation, heat treatment, and storage in semidry fermented sausage. Similar to our findings, Bozkurt (2006) stated gradual increases and decreases in TBARS values of sumac extract and BHT added sucuk samples during fermentation and ripening due to the decomposition of formed TBARS into volatile compounds.

**TABLE 4:** Mean TBARS values of fermented sausages (sucuk) during production and storage.

	Storage	Control	E‐1	E‐5	E‐10	AA
TBARS mg malondialdehyde/kg	0 day	0.97 ± 0.19^abB^	0.88 ± 0.07^bB^	0.35 ± 0.04^cC^	0.32 ± 0.03^cC^	1.13 ± 0.03^aC^
	3 days	1.81 ± 0.13^bA^	1.72 ± 0.02^bA^	1.77 ± 0.02^bA^	1.46 ± 0.01^cA^	2.01 ± 0.05^aA^
	End product	0.74 ± 0.22^aB^	1.18 ± 0.44^aAB^	0.79 ± 0.01^aB^	0.71 ± 0.001^aB^	1.30 ± 0.08^aC^
	30 days	0.65 ± 0.01^bB^	0.81 ± 0.08^bB^	0.75 ± 0.05^bB^	1.70 ± 0.23^aA^	1.97 ± 0.13^aA^
	60 days	1.59 ± 0.01^aA^	1.67 ± 0.14^aA^	1.65 ± 0.11^aA^	1.73 ± 0.14^aA^	1.68 ± 0.03^aB^

*Note*: The difference between the mean values (±SD) indicated with different lowercase letters on the same row is significant (*p* < .05). The difference between the mean values (±SD) indicated with different uppercase letters on the same column is significant (*p* < .05). Control: control group; E‐1: 1 mL/kg bay leaf extract added group; E‐5: 5 mL/kg bay leaf extract added group; E‐10: 10 mL/kg bay leaf extract added group; and AA: 500 mg/kg ascorbic acid added group.

### 3.8 Sensory evaluation

**FIGURE 4:**
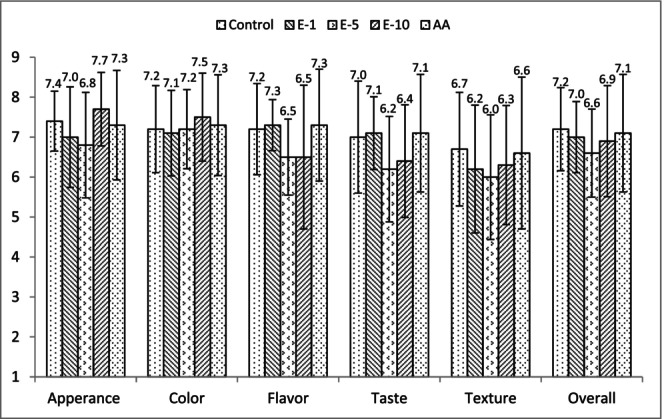
Mean sensory evaluation values of fermented sausages (sucuk). Bars represent standard deviations (SD). Control: control group; E‐1: 1 mL/kg bay leaf extract added group; E‐5: 5 mL/kg bay leaf extract added group; E‐10: 10 mL/kg bay leaf extract added group; and AA: 500 mg/kg ascorbic acid added group.

Sensory evaluation values of fermented sausages are presented in Figure 4. The sensory evaluation of the end product revealed that the treatments had no significant effect on the sensory characteristics of sucuk samples. Sensory characteristics of sucuk samples including appearance scores varied from 6.8 to 7.7, color scores varied from 7.1 to 7.5, flavor scores varied from 6.5 to 7.3, taste scores varied from 6.2 to 7.1, texture scores varied from 6.0 to 6.7, and overall impression scores varied from 6.6 to 7.2. Although E‐5 and E‐10 had numerically lower scores for flavor, taste, and texture, and E1 had numerically higher scores for flavor and taste, the results indicated that the sausages with bay leaf extracts had similar sensory attributes to the control and AA samples and all sucuks were generally liked by the panelists.

## 4 CONCLUSION

The results of this study indicated that bay leaf extract, obtained as a natural antioxidant and a source of phytochemicals, might be effectively incorporated in Turkish fermented sucuks without having a negative impact on their quality attributes or consumer acceptability. The addition of bay leaf extract at levels of 1, 5, and 10 mL/kg into sucuk samples caused no significant decreases in biogenic amine contents in most cases when compared to control and AA samples. Nevertheless, the bay leaf extracts also have no negative effects on the physicochemical, quality, and sensory properties of the sucuks when compared to the control and/or AA samples at the current levels. Thus, bay leaf extract could be commercially utilized for manufacturing fermented sausages with enhanced antioxidant and phytochemical properties. Moreover, the incorporation of bay leaf extracts at higher concentrations or in powder form should also be considered in future studies.

## AUTHOR CONTRIBUTIONS

**Hakan Benli:** Conceptualization (lead); formal analysis (lead); funding acquisition (lead); investigation (equal); methodology (equal); project administration (lead); resources (lead); supervision (lead); validation (equal); visualization (lead); writing – original draft (lead); writing – review and editing (lead). **Pelin Şahin:** Conceptualization (equal); formal analysis (equal); funding acquisition (supporting); investigation (equal); methodology (equal); project administration (supporting); resources (equal); validation (equal); writing – original draft (supporting); writing – review and editing (supporting). **Erdal Ağçam:** Conceptualization (equal); formal analysis (equal); investigation (equal); methodology (equal); resources (equal); validation (equal); writing – original draft (equal); writing – review and editing (equal).

## CONFLICT OF INTEREST STATEMENT

The authors certify that they have no conflict of interest.

## Supporting information


**Figure S1.**.

## Data Availability

The relevant data will be supplied upon reasonable requests.
