# Supplementary material for: Incorporating bay leaf extract (Laurus nobilis L.) and determining the quality attributes of Turkish fermented sausage (sucuk)
Source: Food Sci Nutr. 2024 Jan 2;12(4):2473–87. doi: 10.1002/fsn3.3929 (PMC11016401; doi:10.1002/fsn3.3929)
Supplement: Supplementary file 1 — Figure S1.. [file FSN3-12-2473-s001.docx]

Supplementary Figure 1. A representative HPLC chromatogram of biogenic amines in sucuk sample
